# The Dyad Symmetry Element of Epstein-Barr Virus Is a Dominant but Dispensable Replication Origin

**DOI:** 10.1371/journal.pone.0018609

**Published:** 2011-05-16

**Authors:** Elisabeth Ott, Paolo Norio, Marion Ritzi, Carl Schildkraut, Aloys Schepers

**Affiliations:** 1 Department of Gene Vectors, Helmholtz Zentrum München, München, Germany; 2 Department of Cell Biology (CH 416), Albert Einstein College of Medicine, New York, New York, United States of America; Hannover Medical School, Germany

## Abstract

*OriP*, the latent origin of Epstein-Barr virus (EBV), consists of two essential elements: the dyad symmetry (DS) and the family of repeats (FR). The function of these elements has been predominantly analyzed in plasmids transfected into transformed cells. Here, we examined the molecular functions of DS in its native genomic context and at an ectopic position in the mini-EBV episome. Mini-EBV plasmids contain 41% of the EBV genome including all information required for the proliferation of human B cells. Both FR and DS function independently of their genomic context. We show that DS is the most active origin of replication present in the mini-EBV genome regardless of its location, and it is characterized by the binding of the origin recognition complex (ORC) allowing subsequent replication initiation. Surprisingly, the integrity of *oriP* is not required for the formation of the pre-replicative complex (pre-RC) at or near DS. In addition we show that initiation events occurring at sites other than the DS are also limited to once per cell cycle and that they are ORC-dependent. The deletion of DS increases initiation from alternative origins, which are normally used very infrequently in the mini-EBV genome. The sequence-independent distribution of ORC-binding, pre-RC-assembly, and initiation patterns indicates that a large number of silent origins are present in the mini-EBV genome. We conclude that, in mini-EBV genomes lacking the DS element, the absence of a strong ORC binding site results in an increase of ORC binding at dispersed sites.

## Introduction

**Figure 1:**
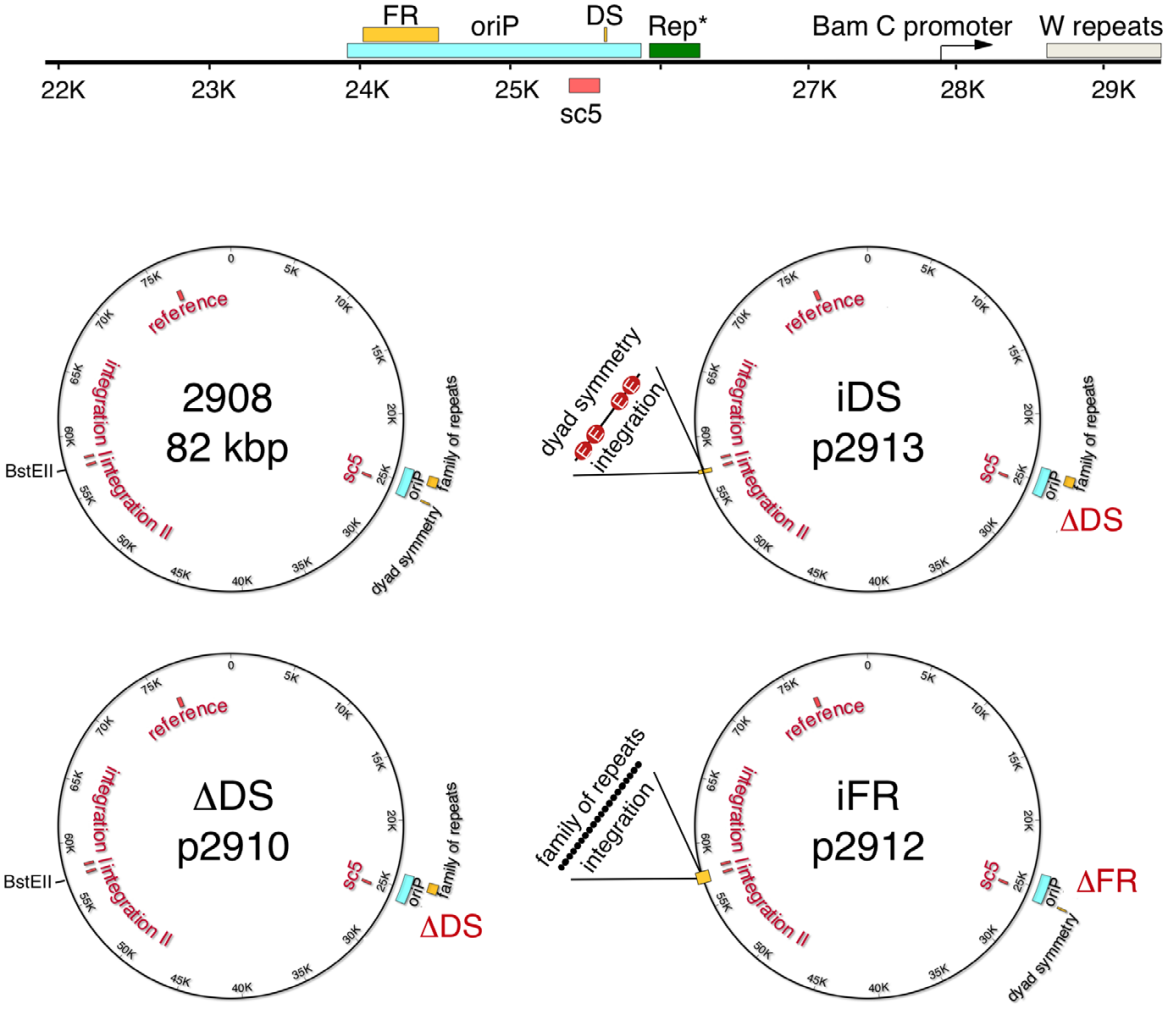
Maps of the mini-EBV plasmids used to transform human primary B-lymphocytes. (A) The latent origin, schematically shown at the top. The minimal *oriP* encompasses the family of repeats (FR) and the dyad symmetry element (DS). Rep* is a 298 bp fragment that can partially substitute the DS-element if multimerized on a plasmid [54]. The *oriP*-specific PCR fragment sc5 (red box) was utilized for quantification experiments. Depicted cis- elements are *oriP*, the *Bam*C promoter and the W repeats (grey box). (B) Starting from p2908, three mutants were generated: ΔDS(p2910) that is lacking DS and iDS(p2913) and iFR(p2912), with either DS or FR being transferred to the indicated *Bst*EII-site, which is positioned on the opposite site of the mini-EBV genome [36]. The PCR-fragments used for the quantification of the ChIP experiments are sc5 for *oriP*, *integration I* and *II* and the reference site.

EBV infects human B-cells and establishes a persistent latent infection. The viral genome is maintained autonomously in proliferating cells and each viral episome is replicated once per cell cycle during S phase [1], [2]. In contrast to other latently infecting viruses, such as papilloma virus, EBV does not override the cellular replication machinery. Therefore, EBV episomes can serve as a model system for chromosomal replication. *OriP* is a 1.8 KB fragment that was discovered due to its ability to support autonomous replication of small plasmids [3], [4] and it is composed of two separate EBNA1-binding elements (FR and DS; Figure 1A). FR consists of an array of twenty imperfect 30 bp repeats (each containing an EBNA1 binding site) and contributes to the stable maintenance of *oriP*-containing episomes by preventing their loss during cell division. FR functions by tethering plasmids via EBNA1 to human metaphase chromosomes [5], [6], [7], [8], [9]. In the presence of EBNA1, FR ensures the stable retention of *oriP*-episomes with a plasmid loss rate of 3–5% per generation [10], [11], [12]. In contrast, DS contains four EBNA1 binding sites arranged in pairs and is the only part of *oriP* required for DNA replication initiation in small plasmids. The topology of the EBNA1 binding sites and their affinity towards EBNA1 affect the ability of DS to function as an efficient origin. In fact, EBNA1 is able to direct the assembly of the ORC proteins only to the DS [13], [14], [15]. In the viral genome and *oriP*-derived plasmids, ORC binding is the first step in the process of origin licensing [16]. During mitotic exit and G1, Cdc6 and Cdt1 load the Mcm2–Mcm7 (Mcm2–7)-complex onto chromatin to form the pre-RC [17], [18], [19]. During S phase pre-RCs are lost either by the activation of origins or by passing replication forks [20]. These findings led to the hypothesis that *oriP* is regulated similarly to chromosomal origins, an assumption further supported by the observation that small *oriP*-plasmids do not replicate in an Orc2-mutant cell line and their replication is inhibited by the overexpression of geminin [21].

The interaction between the two *oriP*-elements is not clear. The sequences between DS and FR can be deleted and, to a certain degree, extended without affecting replication competence. However, an insertion of 4,600 bp rendered the plasmid inactive by preventing the growth of viable colonies [22]. In addition, these manipulations can alter the copy number of the *oriP*-plasmids [22] and plasmids carrying DS but lacking FR are replicated in an EBNA1-dependent manner, but are not stably retained regardless of their ability to replicate [23], [24], [25], [26]. Therefore, how far the *oriP*-components can be separated on a plasmid and still be able to function remains to be determined.

The replication and retention of small plasmids being entirely dependent on the integrity of *oriP* led to the assumption that *oriP* might be the only active origin in the context of the entire EBV genome. However, large initiation zones have been identified outside *oriP* by two-dimensional (2D-) electrophoresis and by SMARD (single molecule analysis of replicated DNA) [11], [27]. The use of initiation sites outside *oriP* differs between different strains [28] and is consistent with the observation that also ORC-binding is not limited to *oriP* [29]. This is compatible with experiments showing that DS is dispensable in transformed cell lines and that other origins of replication are activated within the EBV genome when DS is absent [30]. In addition, while DS is essential in small plasmids, it can be replaced by other eukaryotic sequences [31], [32], or by various fragments of the EBV genome [33]. Due to experimental limitations most studies involving *oriP* have been performed with small plasmids of bacterial origins. In contrast, mini-EBV plasmids are large (71 kb) and contain all the viral sequences required with the exception of the genes required for the lytic phase of growth [35]. In addition, a prokaryotic backbone allows the generation of mutants of essential components of the EBV genome in E. coli. In a recent study, we showed that FR is the essential component of *oriP* for the transformation of primary human B cells, whereas DS is dispensable for the infection process and cell transformation [34]. We also found that DS is dispensable for stable maintenance of the mini-EBV genome as previously shown for the entire EBV genome [11], [30].

In the present study, we analyzed the functionality of DS in the context of mini-EBV episomes. Single molecule experiments showed that DS supports initiation with an efficiency of 89%, which is comparable to the rate found in *oriP*-plasmids [12], [35]. This defines DS as a strong origin of replication. ChIP experiments confirmed that, when DS is present, the specific association of ORC is solely dependent on the replicator DS and does not require any neighboring auxiliary DNA sequences. Initiation events detected using multiple techniques confirmed that, within the mini-EBV genome, DNA replication starts primarily near the DS element even when its genomic location is altered. However, if the viral replicator is deleted, multiple weaker origins are used throughout the mini-EBV episome. Nevertheless, replication remains ORC-dependent and limited to the S phase. We conclude that the loss of a strong origin of replication can be compensated by the activation of multiple weaker replication initiation sites.

## Results

### DS is sufficient for site-specific ORC recruitment

Previous experiments demonstrated that the deletion of DS reduces the specific association of ORC at *oriP* [14], [15]. Here, we examined whether ORC recruitment depends on the integrity of *oriP* or by DS alone. We addressed this question by performing ChIP experiments using different mutant versions of *oriP* in context of the mini-EBV genome (Figure 1) [34]. In the mutant *ΔDS(p2910)* the DS element was simply deleted, while in iFR(p2912) and iDS(p2913) the FR and DS elements were transferred to a *Bst*EII (nucleotide 89,146 of B95; Figure 1.) [34]). The integration site is located at about 40 kbp apart from *oriP*.

**Figure 2:**
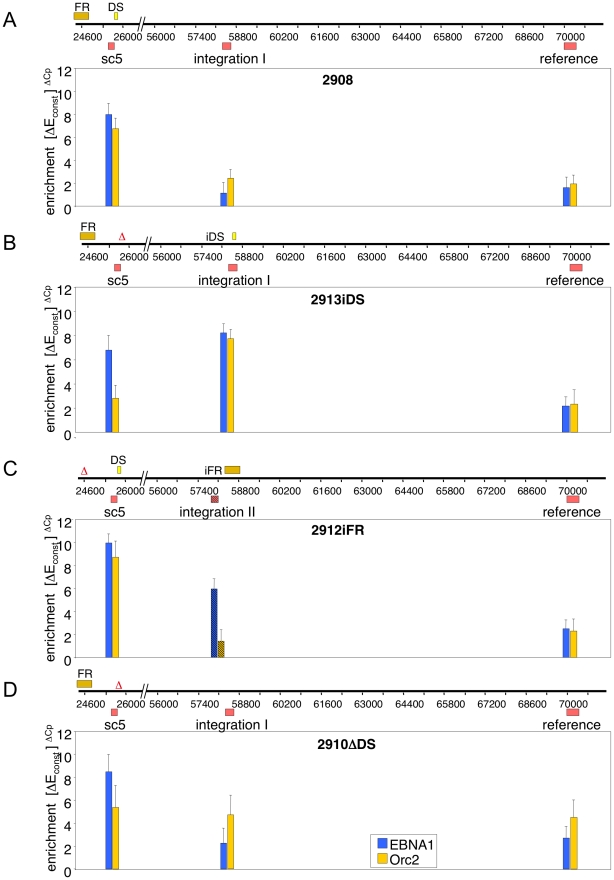
The DS-element is sufficient for site-specific association of ORC. The various mini-EBV mutants were subjected to ChIP-experiments with antibodies directed against EBNA1 and HsOrc2. A linear representation of the mini-EBV genomes is depicted. The different *oriP*-mutants are shown (A–D). FR and the DS are shown as yellow boxes, the deleted element as Δ. The positions and designations of the PCR fragments used to scan the binding of Orc2 and EBNA1 are depicted above the maps and include sc5 (*oriP* region), *integration I* and *II* and the reference site. Crosslinked chromatin of 5×10^6^ cells was isolated and used for each immunoprecipitation and an 1/50 aliquot was used for each PCR. The histogram shows the result of EBNA1 (blue bars) and HsOrc2 (yellow bars). The bars show the difference between the efficiencies (ΔCp) of the specific immunoprecipitates and the isotype controls. The values are represented on the logarithmic y-axis. The graph shows the mean value and standard deviations of at least three experiments. The PCR-fragment *integration II* (striped bar) was used for the mutant iFR(p2912) because the FR could not be amplified by PCR.

Nucleoprotein complexes crosslinked with formaldehyde were isolated after sonication and micrococcal nuclease treatment as previously described [13], [15]. The DNA co-immunoprecipitated with antibodies directed against human ORC2 and EBNA1 was quantified by real-time PCR. The histograms in Figure 2 summarize the results of at least three independent experiments. The antibody used against EBNA1 (blue bars) specifically immunoprecipitated *oriP* in all analyzed mini-EBV genomes (sc5; Fig. 2A–D) [13]. The PCR amplification was up to 100-fold higher compared to the reference site, which is consistent with our previous reports [13], [15]. In contrast, the ectopic integration site was efficiently immunoprecipitated only when FR or DS were present at this location. In iDS(2913), the enrichment at integration *I* (overlapping the integration site) was found to be similar to the enrichment found at *oriP* (Fig. 2B). Hence, EBNA1 binds DS with a similar efficiency in both the wild-type and in the mutant context. In iFR(p2912), we could not amplify the integration sites because the repetitive nature of the FR element. Therefore, we PCR-quantitated an adjacent portion of the mini-EBV genome which is located 0.6 kb upstream of the integration site (integration II, dashed blue line Figure 2C). We found that the amplification level of this genomic region was comparable to integration I in iDS(p2913). Therefore, EBNA1 binds to DS and FR independently from the integrity of oriP.

To test the requirements for ORC-recruitment, we studied the association of human Orc2 in the mini-EBV genome. As expected, ChIP-experiments confirmed that Orc2 is highly enriched at the *oriP* in wild-type p2908 mini-EBV genomes (compare fragment sc5 to *integration I* and to the reference site; Fig. 2 A). Transferring the viral replicator DS to an ectopic integration also increased Orc2 binding to this portion of the genome to about by the 10–20-fold (iDS(p2913), *integration I* in Fig. 2B), which is similar to the level measured at *oriP* in the wild-type mini-EBV genome (yellow bars). In addition, in iDS(p2913), Orc2 binding to *oriP* was reduced to reference level. Hence, DS is mainly responsible for the association of the ORC complex with *oriP*. In contrast, transferring FR to an ectopic position neither reduced the association of Orc2 to *oriP* nor increased ORC-binding to the integration site (*integration II*; iFR(p2912), Fig. 2C). Thus, results indicate that DS is solely responsible for a site-specific binding of ORC.

It is also important to point out that the results obtained in ΔDS(p2910) appear to suggest that the specific binding of ORC to the DS reduces the background level of ORC association to the rest of the mini-EBV genome. In ΔDS(p2910) we found comparable levels of Orc2 binding at all the sites we tested (Fig. 2D), but these levels were higher compared to the background levels of binding found in p2908. Taken together, these initial experiments indicate that DS efficiently recruits Orc2 and the other components of ORC to the mini-EBV genomes. This recruitment is independent of the location of DS relative to FR or to the presence of other auxiliary elements like the Rep*-element [38].

### Cell cycle-dependent formation of pre-RCs at DS

**Figure 3 pone-0018609-g003:**
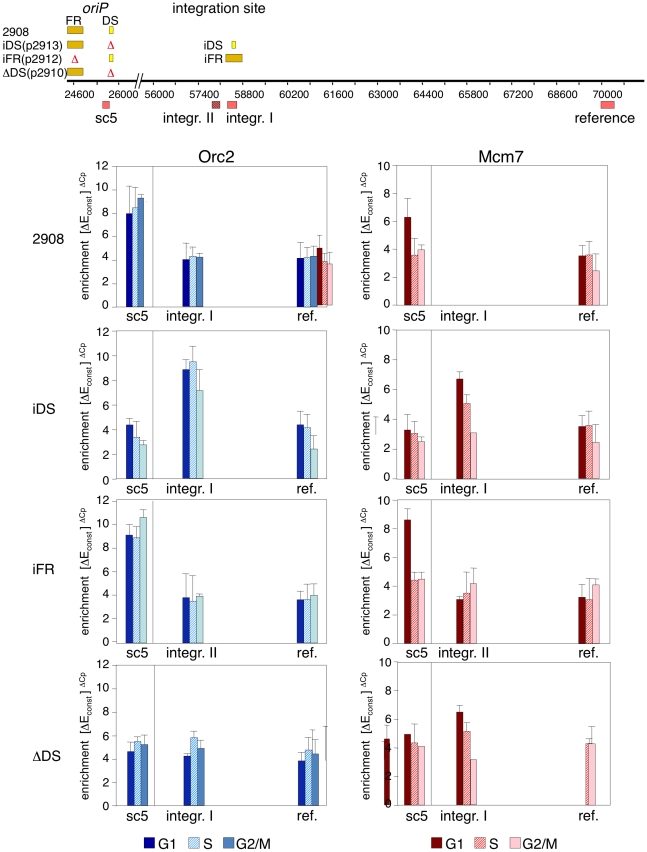
Protein:DNA dynamics of replication proteins at the DS-element of *oriP*. Cell cycle-dependent protein interactions at *oriP* and the integration site were determined by ChIP with antibodies specific to Orc2 (left, blue) and Mcm7 (right, red). A schematic representation of the different mutants is shown on top. Different cell cycle phases were separated by centrifugal elutriation and three fractions were subjected to ChIP (G1 (40 ml minute^−1^), S (60 ml minute^−1^), and G2/M (90 ml minute^−1^)) and immunoprecipitations were analyzed by real-time PCR with primer pairs specific for *oriP* (sc5), the integration site (*integration I* (p2908, iDS(p2913), and ΔDS(p2910)); or *integration II* (iFR(p2912))) and the reference site. The graphs show the mean and standard deviations of at least three independent experiments.

In order to analyze the cell cycle dynamics of pre-RC assembly in various regions of the mini-EBV genome, we performed ChIP in cells cell-cycle fractionated by centrifugal elutriation [4]. After monitoring the fractions by FACS analysis (data not shown), three fractions of the cell cycle (G1, S and G2/M) were analyzed by ChIP using antibodies directed against Orc2 and Mcm7 (Fig. 3). In p2908 (wild-type *oriP*), Orc2 is bound to *oriP* throughout the cell cycle, whereas Mcm7 accumulates during G1 and is released from *oriP* during S phase. This is similar to the results we previously obtained for replication initiation proteins in the prototype mini-EBV strain 1478.A [13]. In addition, we found that the binding of Orc2 to the integration site was also cell cycle-independent and comparable in level to the reference site. The integration and reference sites also shared similar dynamics of Mcm7 binding. Therefore, at least part of the Orc2 and Mcm7 binding to the mini-EBV episomes does not depend on sequence-specific interactions with the DNA. Table 1 summarizes the sequence-specific enrichments of Orc2 and Mcm7 at *oriP* and the integration site.

**Table 1:** Table summarizes the specific enrichment of HsOrc2 and HsMcm7 at *oriP* and the integration site shown in the histograms in Figure 3.

	x-fold enrichment above average of reference level							
	sc5 (oriP)				integration site			
	G1	S	G2/M	average	G1	S	G2/M	average
**HsOrc2**								
2908	16.2	17.2	28.9	20.8±7.1	0.9	1.0	1.0	1.0±0.1
ΔDS(p2910)	1.3	2.3	2.0	1.9±0.5	1.0	2.2	1.5	1.6±0.5
iFR(p2912)	21.8	19.2	35.1	25.4±8.5	1.0	0.9	1.0	1.0±0.1
iDS(p2913)	1.4	1.0	0.8	1.1±0.3	29.8	34.6	16.4	26.9±9.4
**HsMcm7**								
2908	10.6	0.9	1.2	cell cycle	1.8	0.9	0.8	1.2±0.5
ΔDS(p2910)	1.0	1.0	1.0	1.0	1.7	1.0	1.7	1.5±0.4
iFR(p2912)	29.8	2.0	2.1	cell cycle	1.0	1.1	1.6	1.2±0.3
iDS(p2913)	1.4	1.1	0.9	1.1±0.2	13.9	6.5	1.0	cell cycle

The average enrichment at *oriP* and the integration site are normalized in relation to the reference site. Three independent experiments are shown for the three cell cycle fractions analyzed. For location of the PCR primer pairs see Figure 2. Because DS does not exhibit cell cycle-dependent binding of HsOrc2, the mean value and standard deviation of the fragments was used to establish the average enrichment (average). The mean value at the reference site, which represents the non-sequence specific DNA-binding activity of the analyzed protein, was determined and used for normalization to calculate the accumulation for *oriP* and the integration site. The obtained numbers represent the enrichment at the particular fragment above the reference site level. With the exception of ΔDS(p2910), the enrichment of HsMcm7 is cell cycle-dependent (cell cycle). Therefore no average value was determined.

To account for this sequence-independent binding activity of the pre-RC components, we divided the enrichment obtained at *oriP* and at the integration site by the mean enrichment obtained at the reference site. This allowed us to calculate the fraction of Orc2 and Mcm7 binding that depends on sequence-specific interactions (Table 1). Results obtained with iFR(p2912) and iDS(p2913) confirmed that DS is necessary and sufficient for the site-specific formation of pre-RCs to a level comparable to p2908 and 1478.A [13]. The cell cycle-dependent association of Mcm7 is indicative of licensing the mini-EBV episomes during the G1 phase. Comparing the different cell cycle phases indicated a temporal enrichment at DS. The enrichment of Orc2 at or near DS was similar in all three mini-EBV mutants (Table 1). The Mcm7-specific antibody precipitated G1 phase chromatin of the viral replicator 10.6 times as efficient as at the reference site. During later cell cycle phases, no DS-specific enrichment was observed, indicating the release of the Mcm2–7-complex (Fig. 3; Table 1).

The experiments presented above suggest that *oriP* integrity is not required for pre-RC assembly at the DS element. However, the DS replicator is neither essential for the transformation of human primary B-cells nor for stable episomal maintenance of the EBV genome [14], [15], [30]. Cell cycle experiments with ΔDS(p2910) confirmed that Orc2 binds to all tested sites at a comparably low level (Fig. 3, and Table 1), suggesting a diffused and non-linear binding throughout the mini-EBV genome. In addition, site-specific association of Mcm7 was also lost in ΔDS(p2910), but remained detectable at a low level throughout the episomes. In addition, we found that the background levels of Orc2 and Mcm7 binding increase throughout the episomes in ΔDS(p2910) suggesting an increased licensing at several auxiliary initiation sites. This is consistent with previous observations showing that DS-deletion in P3-ΔDS-47 (a P3HR1 strain) results in loss of ORC binding and origin activity at *oriP* and increased origin activation at additional distant sites [15], [30].

### DS-independent replication is limited to once per cell cycle and requires ORC

**Figure 4 pone-0018609-g004:**
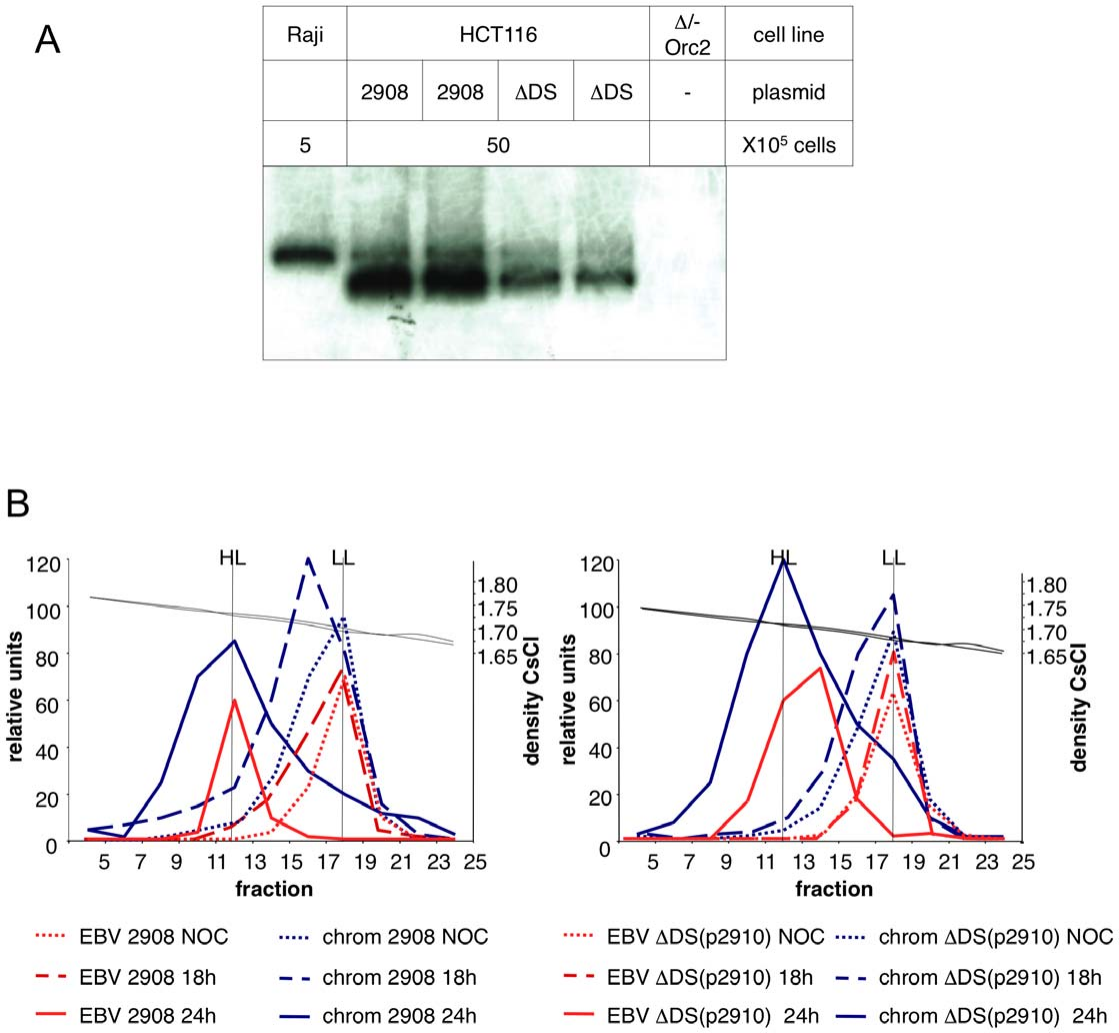
The DS-element is not required for ORC-dependent replication during S phase. (**A**) HCT116 cells were stably transfected with the wild-type *oriP* p2908 and the DS-deletion ΔDS(p2910). The episomal status of single cell clones was determined by Gardella gel analysis. To estimate the copy number of the mini-EBV genomes, the designated number of cells was analyzed per lane. Raji was used for standardization [55]. (**B**) The ΔDS-LCL ΔDS(p2910) replicates once per cell cycle during S phase. Both cell lines were synchronized in mitosis with nocodazole. After release the DNA was labeled with BrdU for 18 h and 24 h, when cells were in late S and mitosis respectively. Total DNA of 1×10^7^ cells was separated on a CsCl gradient. The relative concentration of unsubstituted (LL) and hemisubstituted (HL) DNA respectively was determined by real time PCR using mini-EBV (red lines) and the bulk chromosomal DNA (blue lines) and plotted against the CsCl-density. The densities of LL and HL DNA 1.70 and 1.73 g/cm^3^ are indicated on the right of the graphs.

*OriP*-driven replication is known to depend on ORC binding, since small *oriP*-plasmids cannot be established in ORC2Δ/- hypomorphic cells and DS-mediated replication is inhibited by geminin [36]. Are the auxiliary origins used in ΔDS(p2910) also depending on ORC and pre-RC assembly? To test this possibility, we performed transient replication experiments of ΔDS(p2910) and p2908 episomes in the ORC2Δ/- hypomorphic cell line e83 and its parental cell line HCT116 [21]. Cells transfected with either p2908 or ΔDS(p2910) were selected using the hygromycin selection marker. Hygromycin resistant cell clones were easily obtained for the HCT116 cell line with both mini-EBV genomes. The efficiency of colony formation in HCT116 cells after two weeks of selection was similar for both p2908 and ΔDS(p2910) (6.65% and 5.55%, respectively; see Table S1). In addition, after four weeks of selection the episomal status of the mini-EBV genomes was also confirmed by electrophoresis in Gardella gels (Fig. 4A) confirming that both types of episomes can efficiently replicate in HCT116 cells. In contrast, both p2908 and ΔDS(p2910) failed to generate stable hygromycin-resistant clones in ORC2 Δ/- cells. The inability to establish stable episomal replication in ORC2-hypomorphic cells suggests that the maintenance of both genomes depends on either one or few inefficient origins per episome and that the intracellular concentration of ORC proteins is crucial for the efficient replication of mini-EBV episomes regardless of the origins being used (*oriP* or auxiliary origins).

To confirm that ΔDS(p2910) replicates only once per cell cycle we performed a density transfer assay according to Meselson-Stahl. HCT116 cells stably transfected with p2908 or ΔDS(p2910) were synchronized in mitosis using 2 mM nocodazole for 10 h. Mitotic cells were isolated by shake-off and released from the cell cycle block by transferring them into fresh culture medium containing 5-bromodeoxyuridine (10 µM BrdU), which is incorporated in the replicating DNA increasing its buoyant density. Following labeling periods of 18 and 24 hours, the total genomic DNA was then extracted, digested with *Bam*H1, and fractionated by equilibrium centrifugation on CsCl gradients to measure the incorporation of BrdU in the cellular DNA and in the mini-EBV genomes. The positions of the mini-EBV sequences within the gradients were determined by real-time PCR and compared to the position of bulk chromosomal DNA. The relative concentrations of these two DNA populations is shown in Fig. 4B. Results clearly indicate that both p2908 and ΔDS(p2910) replicate once per cell cycle. However, these two mini-EBV episomes replicate with different efficiencies. Twenty-four hours after releasing the cells from the nocodazole block, nearly all the chromosomal DNA and the p2908 mini-EBV DNA have shifted from the light LL form (unsubstituted) to the heavy–light HL from (hemisubstituted) indicating the completion of the replication cycle. In contrast, the ΔDS(p2910) DNA is still partially unreplicated indicating that DNA replication initiation is inefficient in the absence of the DS element.

### Detection and analysis of replication intermediates

**Figure 5 pone-0018609-g005:**
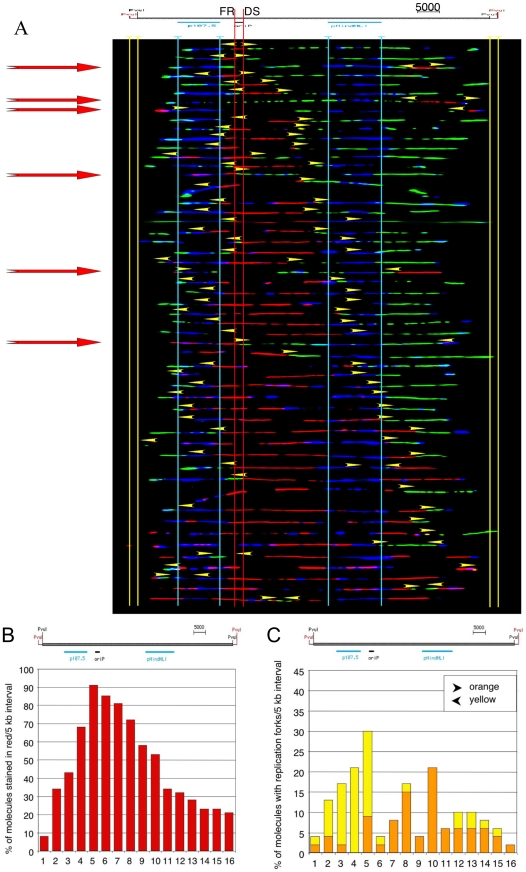
Mapping of replication initiation events by single molecule. (**A**) On top is a map of the *Pvu*I-linearized mini-EBV genome with positions of *oriP* indicated in black and the position of the hybridization probes utilized to detect the DNA molecules of interest, which are indicated in blue (to scale). Vertical red lines show the position of *oriP*, the yellow lines of the *Pvu*I sites. The results of SMARD are presented as a series of images of DNA molecules representative of the different stages of duplication of a specific genomic region. Aligned to the maps are the images of the red and green molecules organized from top to bottom by increasing content of IdU (red). Yellow arrowheads mark the positions of the red-to-green transitions along the molecules (corresponding to the positions of the replication forks at the end of the first labeling period). Most of the forks appear to originate form *oriP*. Some molecules however clearly used alternative initiation sites (red arrows). (**B**) The results of SMARD are also presented as a summary for the entire population of molecules (here called replication profile) shown below the schematic outline of the molecules. The horizontal axis of the diagram represents arbitrary ∼5 kb intervals of the mini-EBV episome. The vertical axis shows the percentage of red-green molecules stained in red. The intervals more frequently stained in red are those that replicate first on average. The profile shows a clear peak at *oriP*. (**C**) The profile of replication fork abundance shows the percentage of molecules that contained a replication fork at the time of label switch at each position along the restriction fragments. Left-moving and right-moving forks are shown separately in yellow and orange bars respectively.

The dominant but not exclusive initiation of DNA replication in proximity of DS was confirmed by three independent mapping approaches. The nascent strand abundance assay quantifies small 0.8–1.3 kb DNA-fragments emerging from active origins of replication. This approach showed that a peak of nascent DNA is visible in proximity of the DS element (Fig. S1). This result was confirmed by 2D-gel electrophoresis, which shows the presence of a complete bubble arc at *oriP* (Fig. S2) and by SMARD. These data indicate that *oriP* is the strongest initiation sites present in the mini-EBV episomes (Fig. 5A, B). In addition, SMARD allowed us to detect the occurrence of initiation events at additional locations in the episomes, although at low resolution compared to 2D-gel and nascent strand analysis. Six out of 53 of the replication intermediates detected by SMARD showed the presence of initiation events clearly occurring outside *oriP* (∼11%). This suggests that initiation events occur with much lower efficiency at these sites compared to *oriP*. In addition, the replication profile of the episomes indicates that replication forks pause or terminate at FR (Fig. 5C; Fig. S2), which is in line with previous studies [11], [37]. This suggests that deletion of the DS results in the utilization of alternative origins whose activity is normally overshadowed by initiation in proximity of DS. Hence, *oriP* is the dominant initiation start site in context of the mini-EBV genome.

### The DS is required for site-specific initiation

**Figure 6:**
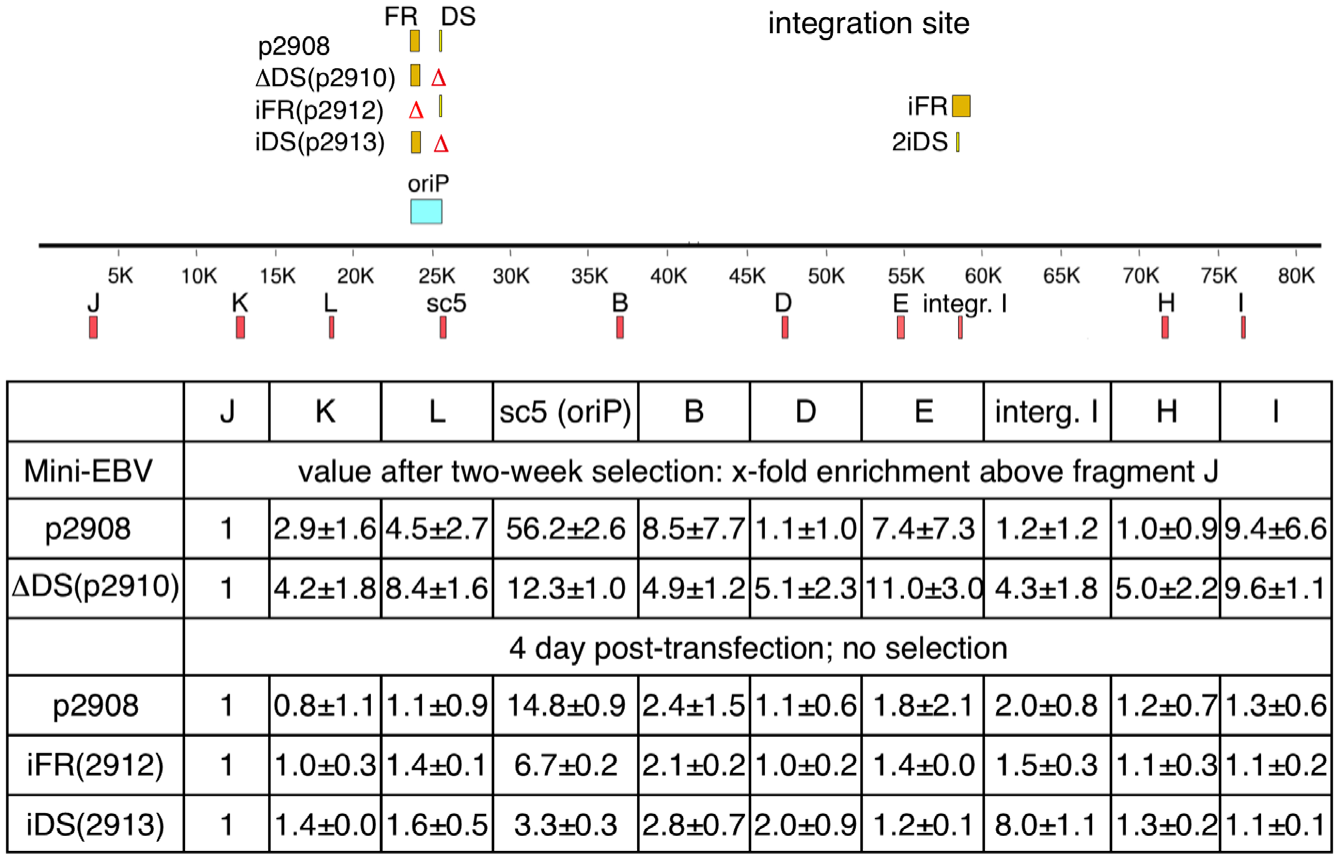
Nascent strand abundance of the mini-EBV episomes in HCT116 cells. A view of the mini-EBV genomes is shown on top. The table summarizes the x-folds of enrichment for the nascent strand abundance using fragment J as reference. Fragment J, which does not bind components of the replication initiation machinery [15], is located in the prokaryotic backbone of the mini-EBV episomes as shown in the schematic view of the mini-EBV genome together with the location of the different amplified regions. The enrichment and standard deviation of at least three independent experiments is shown. **(Top lines)** Nascent strand DNA of p2908 (wild-type *oriP*) and ΔDS(p2910) were determined in HCT116 cells after two weeks of selection. Both genomes are episomally stably maintained and replicate in an ORC-dependent manner. **(Lower lines)** Nascent strand abundance of HCT116 cells transiently transfected with the mini-EBV episomes p2908 (wild-type *oriP*), iFR(p2912) and iDS(p2913). Nascent strand abundance was analyzed 96 h post transfection and without supplying selective pressure. The nascent strand abundance of each PCR-fragment was normalized in relation to the non-origin PCR-fragment J.

The experiments described above indicate that DS is necessary and sufficient for the association of ORC to *oriP*. Does this result in site-specific initiation of DNA replication? To answer this question, we first determined whether the deletion of DS alters the accumulation of nascent DNA strands at *oriP*. Toward this end, we compared *oriP* activity in p2908 and ΔDS(p2910) episomes stably transfected in HCT116 cells. In p2908, initiation events occur near *oriP* as they do in the prototype mini-EBV genome 1478.A replicating in LCL A39 cells (Fig. 6 and Figs. S1 & S2). Hence, the overall initiation pattern is cell type independent. Only marginal differences were observed between different cell lines, indicating that genetic elements or the epigenetic status of the mini-EBV genome might influence the initiation pattern. In contrast, in ΔDS(p2910) initiation events, were distributed over the entire genome (Fig. 6). The levels of nascent strand at sc5 were still higher than at other regions of the mini-EBV genome. This indicates that region-specific characteristics, such as an open chromatin structure, might facilitate replication initiation. However, these differences were not as dominant as the presence of DS.

To determine if DS is responsible for site-specific initiation we also analyzed the *oriP*-mutants iFR(p2912) and iDS(p2913) in transient assays. For this purpose we made use of HCT116 cells transiently transfected with the different *oriP*-constructs. Nascent strands were analyzed 96 h post transfection. Control experiments with the wild-type mini-EBV p2908 show that nascent strand DNA is, on average, 10-fold enriched at *oriP* than in regions other than *oriP* (Fig. 6, lower part). These transient assays facilitate the mapping of initiation events and demonstrate that shortly after transfection the same initiation sites are used as in established cell lines with stably replicating episomes. Independent experiments using iFR(p2912) and iDS(p2913) confirm that the DS recruits ORC and other pre-RC proteins site-specific and that is also the preferred initiation site (Fig. 6). Initiation events are three- to four-fold more frequent at DS compared to other sites. No other replication origin is activated with high frequency, if DS is present, whereas no dominant initiation site is detected if DS is absent.

## Discussion

The replicative function of *oriP* has been subject of numerous studies based on various experimental systems. The use of different experimental systems has sometimes generated contradictory results. For example, while small *oriP*-plasmids are entirely dependent on DS for their replication, viral DNA synthesis in Raji cells is independent of DS (less than 10% of the initiation events take place at or near DS) [11], [27], [28], [29]. Hence, for this study we utilized mini-EBV episomes to analyze if the integrity of *oriP* or DS alone contributes to replication competence and origin usage in one experimental system.

### DS is a dominant replicator and is sufficient for site-specific ORC-binding and replication initiation

It is becoming increasingly clear that both genetic and epigenetic features of the genomic context can affect the maintenance efficiency of *oriP*-based plasmids resulting in different copy numbers. In this report we studied the efficiency of pre-RC assembly and site-specific replication initiation in the context of mini-EBV genomes. Toward this end, we spatially separated DS and FR and also deleted DS. Our data provide evidence that in mini-EBV genomes DS is the predominant site of ORC-binding and pre-RC assembly and also the preferred initiation start site regardless of the cellular background (Figs. 2 to [Fig pone-0018609-g003] [Fig pone-0018609-g004] [Fig pone-0018609-g005] 6 and S1, S2). Nascent strand analyses provide evidence that DS is predominately used in LCLs bearing the wild-type mini-EBV genome as well as in HCT116 cells stably transfected with the wild-type mini-EBV p2908 (Figs. 5 and 6). The spatial separation of DS and FR in iFR(p2912) and iDS(p2913) result in a slightly more relaxed initiation pattern, although DS is still the most active initiation start site. The initiation efficiency of a single replication origin in relation to other origins is indicative for its dominance. Previous ChIP-experiments have demonstrated that in wild-type EBV-genomes human ORC and MCM proteins bind to *oriP* and that pre-RCs are mainly assembled at DS [13], [14], [15], [21]. A mutational analysis indicated that at least one intact pair of EBNA1 binding sites is required for pre-RC formation [38]. In addition, from previous experiments it is known that ORC and other pre-RC components could be targeted to DS in context of *oriP*-plasmids and EBV-genomes via EBNA1 [3], [13], [14], [15], [21]. Transferring DS in iDS(p2913) or FR in iFR(p2912) to another position of the mini-EBV genome now provides conclusive evidence that this 114 bp-element is sufficient for the site-specific association of ORC and the cell cycle-regulated assembly of pre-RCs. Whereas the efficiency of pre-RC-formation at DS was not affected by its genetic context (Figure 3), the initiation pattern was slightly more relaxed (Figure 6). Although DS is still the preferred initiation start site, these findings suggest that the epigenetic and/or genetic environment is/are not important for pre-RC formation but can influence the initiation efficiency to a moderate degree.

### Deletion of the dominant replicator leads to the activation of silent origins

The translocation experiments demonstrated by ChIP and initiation start site mapping that DS is the predominately active origin in context of mini-EBV genomes. However, the DS-deletion mutant ΔDS(p2910) has a completely different ORC-binding and initiation pattern. This observation suggests the existence of a hierarchical order of strong dominant origins and, of an abundant number of rarely used weak auxiliary origins of DNA replication [39]. This phenomenon is called origin interference and examples are described in viral as well as cellular systems. Auxiliary origins are not very active in mini-EBVs containing DS (Figs. 5 and 6). For unknown reasons, these usually silent origins are more active in some EBV genomes such as in Raji cells (the initiation zone mapped in the Raji genome is absent in the mini-EBV genome) [11], [27]. In this study, we provide evidence that in the absence of DS, replication of the mini-EBV genome is still dependent on ORC and follows the once-per-cell cycle rule. In HCT116 cells, the mini-EBV genomes wt-*oriP*p2908 and ΔDS(p2910) are autonomously maintained, whereas extrachromosomal copies are not established in the Orc2-hypomorphic HCT116 cell line e83 (Fig. 4A). Hence, active origins can be defined in both a DS-dependent and in a DS-independent manner. In case of the mini-EBV genome, initiation at DS is dominant. The predominance of DS-mediated replication is less pronounced in full size EBV genomes. The deletion of DS results in a loss of *oriP*-specific binding of replication initiation proteins, which leads to an increased loss of replication competence in case of very small plasmids, but is tolerated in case of large EBV genomes [14], [15]. Similar to the findings by M. Calos and colleagues, who reported that eukaryotic DNA segments >15 kb replicate with similar efficiencies in a once per cell cycle manner in cultured cells, our results indicate that plasmid-size matters. Whole and mini-EBV genomes are sufficiently large to support long-term autonomous replication from multiple locations on the plasmid [40], [41]. The non-linear usage of only one or very few of many possible silent origins in a single ΔDS-genome has been discussed before for the episomal pEPI-vector [42]. In a recent study, Wang and Sugden compared the role of DS and the Raji-origin in establishing newly transfected plasmids and in maintaining established plasmids [33]. The authors argue that DS is essential for the establishment, whereas other EBV sequences can support DNA synthesis of established plasmids. Our experiments using the mini-EBV system demonstrate that DS is non-essential in establishing mini-EBV genomes transduced via infection [34]. However, the integrity of *oriP* facilitates higher copy numbers (ibid).

The presence of auxiliary origins in the EBV genome is consistent with the observation that in Raji and other Burkitt's lymphoma cell lines the binding of pre-RC components is also not restricted to *oriP* [27], [28]. However, previous results have also shown that in mini-EBVs ORC-binding appears to be restricted to DS [13], [15]. In this study, we extended these findings showing that the deletion of a discrete replicator, like DS, leads to increased binding of ORC and pre-RC proteins at different locations in the mini-EBV genome. These findings could potentially explain the much more evenly distributed initiation pattern in ΔDS(p2910) (Figs. 4 and 6). Wild-type EBV genomes, however, are much larger than mini-EBVs and significant numbers of pre-RC could be assembled at alternative sites during every duplication cycle.

One example for origin interference at chromosomal regions is the Dehydrofolate Reductase (DHFR) origin of replication. The 50 kb spanning DHFR locus contains three preferred initiation sites. When all three initiation sites are deleted initiation still occurs but at many other sites in the non-transcribed spacer region with lower frequency [43]. It is worth noting that initiation sites are not observed in sequences within the DHFR gene. However, these sequences, when transferred to another site, can serve as initiation sites [44]. Thus, several lines of evidence indicate that the genetic and epigenetic contexts play an important role in specifying and selecting initiation sites in mammalian cells. In addition, there are likely to be other yet unknown factors. Such a plastic initiation pattern is also present in the mouse immunoglobulin heavy-chain locus [45].

Our results suggest that many viral origins may actually be located in replication initiation zones. A study has shown that, surprisingly, DNA synthesis generally initiates outside the simian virus 40 core origin *in vitro* [46]. Further, we have shown that DNA synthesis in the Raji EBV genome initiates within a replication initiation zone, which is influenced by its genomic context [11], [27], [28]. Numerous studies on replication initiation zones have been performed in mammalian cells. However, it is still not understood how replication timing as well as initiation can be very specific and highly regulated and yet apparently not dependent on specific replication initiation sequences. We believe that the EBV replication system can shed light on this conundrum.

In the present study, we have provided new insights into how the chromosomal context surrounding DS affects initiation. DS specifies the dominant site of pre-RC assembly in whole and mini-EBV genomes and is a strong initiation site in the mini-EBV system. We show that, to function, DS is not required to be in the context of *oriP*. As long as the DS is present, alternative initiation sites are not efficiently activated. However, if DS is absent, replication initiates from normally silent origins in the mini-EBV genome. These alternative weak EBV origins, might be similar to inefficient mammalian origins [47]. The question of what constitutes mammalian origins, is still unclear [39], [48]. Generally, only a subset of replication competent origins established in G1 fire in S phase [49]. Previous approaches to isolate and characterize such weak initiation sites proved to be very difficult. One reason is that each of these weak origins is probably not frequently active in a plasmid context and does not function as a high-efficient autonomously replicating sequence [50]. For example, in the initial screen that led to the identification of *oriP*, the entire EBV genome was scanned for episomal replication activity, but only the *oriP*-fragment supported this autonomous replication [4]. We could recently show that only a part of strong chromosomal origins are active in *oriP*-based plasmids [51]. Further studies are required to address the question, how the plasticity of origin activation is regulated in different EBV strains [28]. This would also address the question, whether the frequency of origin activation is based on an active process or depends on non-linear origin usage.

The results of the ChIP-experiments presented in this study indicate that the association of ORC and the regulated formation of the pre-RC in G1 are solely dependent on DS and not on its genomic context. Hence, DS possibly provides a targeting site for pre-RC formation, which is not dependent on a specific genetic or epigenetic context. In contrast, the formation of pre-RCs at other chromosomal locations could be more sensitive to the genomic context. In particular, their assembly could be inhibited by the assembly of pre-RCs at adjacent chromosomal positions, as previously suggested for the *in vitro* replication systems based on Xenopus egg extracts [52]. Origin interference might not only occur at the level of pre-RC formation, but also at the level of selection of initiation start sites. Nascent strand analysis indicates that initiation is more relaxed in mini-EBV mutants with separated *oriP*-elements than in their native contexts. Therefore, the formation of a pre-RC is not the only criterion to describe and explain origin activity. The surrounding genetic and epigenetic elements could also separately affect the efficiency of origin activation.

## Materials and Methods

### Cell culture

A39 is a lymphoblastoid B-cell line generated from human primary B-lymphocytes with EBV virions containing the mini-EBV 1478.A [13], [15]. The LCLs p2908 ΔDS(p2910), iFR(p2912), and iDS(p2913) were maintained in RPMI1640-medium supplemented with 10% fetal calf serum (Biochrom) as described [34]. The colon carcinoma cell line HCT116 and the ORC2-hypomorhic cell line e83 were maintained in McCoy's medium complemented with 10% FCS [36]. Mini-EBV plasmids were transfected with Polyfect according to the manufacturers instructions and selected with hygromycin (HCT116: 50 µg/ml; e83: 25 µg/ml).

### Centrifugal elutriation and flow cytometry

Centrifugal elutriation (Beckman J6-MC centrifuge) was used to separate the different cell cycle phases. For chromatin immunoprecipitation experiments 5×10^9^ logarithmically growing LCLs were washed with PBS and resuspended in 50 ml HBSS supplemented with 1% FCS, 1 mM EDTA and 0.25 U/ml DNase I (Roche). Cells were injected in a JE-5.0 rotor with a large separation chamber at 1500 rpm and a flow rate of 30 ml/minute controlled with a Cole-Palmer Masterflex pump. The rotor speed was kept constant and 400 ml fractions were collected at increasing flow rates (35 to 100 ml/minute). Individual fractions were counted and processed for the chromatin immunoprecipitation assay as described below.

### Chromatin-immunoprecipitation assay and PCR analysis

For chromatin-immunoprecipitation experiments, 1×10^7^ nuclei were prepared for each immunoprecipitation as described before [13]. For each immunoprecipitation 1×10^7^ cells were harvested, washed with PBS and resuspended in 250 µl hypotonic buffer A (10 mM Hepes pH 7.9, 10 mM KCl, 1.5 mM MgCl_2_, 0.34 M sucrose, 10% glycerol, 1 mM DTT, protease inhibitor mix Complete^©^ (Roche)). Cells were lysed by adding 0.04% Triton X-100 and incubated for 10 minutes on ice. Samples were centrifuged (4 minutes, 1300 g, 4°C) to separate soluble cytosolic and nucleosolic proteins from chromatin. Nuclei were washed at a concentration of 1×10^8^ nuclei/ml in ice-cold buffer A supplemented with 200 mM NaCl. After centrifugation (1300 g, 5 minutes, 4°C) nuclei were carefully resuspended in 1 ml buffer A. 9 ml pre-warmed buffer A supplemented with 1.1% formaldehyde were added and the nuclei were cross-linked for 10 minutes at 37°C. Fixed nuclei were washed twice with PBS/0,5% NP40, resolved in 2.7 ml LSB (10 mM Hepes pH 7.9, 10 mM KCl, 1.5 mM MgCl_2_) and lysed by adding 300 µl 20% Sarkosyl. The chromatin was transferred onto a 40 ml sucrose cushion (LSB; 100 mM sucrose) and centrifuged (10 minutes, 4°C, 4000 g). Supernatant was removed and the chromatin was resuspended in 2 ml TE and sonicated (Branson sonifier 250-D, 35% amplitude, 2 minutes in 1 second intervals). For partial DNA digests, 2 mM CaCl_2_ and 8 U MNase (Roche) were added to the chromatin and incubated for 10 minutes at 37°C. Adding 5 mM EGTA stopped the reaction.

For immunoprecipitation, the extract was adjusted with 1/10 volume of 11× NET (0,5 M Tris, 1.5 M NaCl, 0.5 M EDTA, 5% NP40). 10 µg affinity purified polyclonal antibodies (Orc2, Mcm7), or 50 µl supernatant of the monoclonal EBNA1-specific antibody were added respectively. The immunoprecipitation and purification of co-precipitated DNA was performed as illustrated [15]. Real-time PCR analysis was performed according to the manufacturers instructions using the same parameters and primer pairs as described [15]. A detailed chromatin immunoprecipitation protocol is available at: http://haema145.gsf.de/.

### Separation of BrdU-substituted DNA

HCT116 cells carrying ΔDS(p2910) or p2908 were synchronized for 10 h with 2 mM nocodazole, isolated by mitotic shake off and transferred to a cell culture medium containing 5-bromodeoxyuridine (10 µM BrdU) to increase the buoyant density of replicated DNA for CsCl equilibrium gradient centrifugation. Total DNA was isolated from 1×10^7^ cells, digested with the restriction enzyme *Bam*H 1 and separated on CsCl gradients adjusted to a refractive index of 1.403. The gradients were spun in a Beckman SW41 rotor at 40,000 rpm for 48 h. Fractions of 250 µl were collected and their refractive index was determined. Samples were diluted, precipitated and further analyzed by quantitative PCR.

### Nascent strand analysis [53]

2×10^7^ cells were washed with PBS and resuspended in PBS with 10% glycerol. Cells were lysed for 10 min in slots of a 1.2% alkaline agarose gel (50 mM NaOH, 1 mM EDTA). DNA was separated by electrophoresis overnight (low melting temperature agarose, Biozym). Fragments of 0.8 to 1.3 kb were extracted using QIAquick gel extraction kit (QIAGEN). Nascent strand abundance was measured by quantitative real-time PCR using the same parameters and primer pairs as for the chromatin-immunoprecipitation experiments [15].

## Supporting Information

Figure S1 **The abundance of short nascent DNA strands at the chromosomal HPRT-locus and the mini-EBV-genome A39 was determined by real-time PCR.** A schematic representation of the mini-EBV-genome is given (top). The locations and designations of the PCR fragments used to scan the nascent strand abundance are shown below the ruler. A standard curve was used for each primer pair to estimate the copy number of nascent strands. The obtained values were normalized for the copy number of primer pairs 7 (HPRT) and sc5 (mini-EBV) respectively, which were arbitrarily set as 100%. The mean values and standard deviations are calculated of four independent experiments. The average value of all PCR-fragments outside *oriP* is shown as a dotted line.
(DOC)

Figure S2 **The diagram on top explains the migration pattern of different replication intermediates expected from 2-D gel analysis, including Y-arcs and bubble arcs.** The dark spot in the lower right is the position where non-replicating monomer-length molecules migrate. Non-replicating linear molecules of different sizes migrate along the arc indicated. Three classes of replicating intermediates are shown in the diagram: The bubble arc showing internal initiation site, the simple Y-arc indicating that the fragment is passively replicated by single fork originated outside the fragment and random termination showing two replication forks converge at multiple sites across the region. (**A**) Analysis of replicative intermediates for the cell line Be302-16 latently infected with a mini EBV genome having 71% of the entire EBV sequence deleted. *Eco*RI and *Dra*I digested nuclear matrix associated DNA (75 to 100 µg) were enriched for replication intermediate through BND column chromatography. After 2-D gel electrophoresis and southern transfer the membranes were hybridized to a 1 kb *Eco*RI-*Mlu*I fragment containing the FR region (B95-8 coordinates #7316 to #8315). On top a scheme of *oriP* is shown including a diagram of the 2-D gel pattern obtained with this cell line. (**B**) Autoradiogram of the hybridized blot. Here we did not see any random termination or accumulation of small mass close to 1× spot indicating that not many forks are coming from outside the segment analyzed, but we observed strong initiation from *oriP*.
(DOC)

Table S1 **Establishment efficiency of p2908 and p2910ΔDS.** 5×10^5^ cells were transfected with 2 µg purified mini-EBV DNA. At 2 days posttransfection, the percentage of EGFP-positive cells was determined and duplicates of 10^4^ and 10^3^ cells were plated per 15-cm dish in media containing 45 µg/ml hygromycin B. After selection for 2–3 weeks, the drug-resistant colonies were counted. These data allowed the calculation of the establishment efficiency using the equation: (No. of drug-resistant colonies/with No. of EGFP-positivel transfected cells)×100%. a) Numbers reflect the average of 2 dilutions of cells plated. b)Transfection efficiency is based upon the percentage of EGFP-positive cells present at 2 days posttransfection.
(DOC)
