# Supplementary figures and images for: The Dyad Symmetry Element of Epstein-Barr Virus Is a Dominant but Dispensable Replication Origin
Source: PLoS One. 2011 May 16;6(5):e18609. doi: 10.1371/journal.pone.0018609 (PMC3095595; doi:10.1371/journal.pone.0018609)

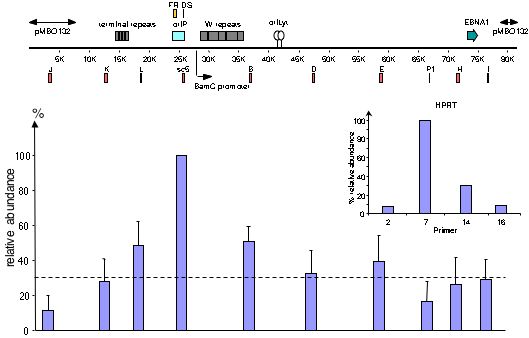


2800

2908

2910ΔDS

2912

2913

.4

.I

.II

.4

.XI

.4

.XII

.3

M

wt

loxP

Δ DS

ectopic DS

ectopic FR

Supplement: Figure S1 — The abundance of short nascent DNA strands at the chromosomal HPRT-locus and the mini-EBV-genome A39 was determined by real-time PCR. A schematic representation of the mini-EBV-genome is given (top). The locations and designations of the PCR fragments used to scan the nascent strand abundance are shown below the ruler. A standard curve was used for each primer pair to estimate the copy number of nascent strands. The obtained values were normalized for the copy number of primer pairs 7 (HPRT) and sc5 (mini-EBV) respectively, which were arbitrarily set as 100%. The mean values and standard deviations are calculated of four independent experiments. The average value of all PCR-fragments outside oriP is shown as a dotted line. (DOC) [file pone.0018609.s001.doc]

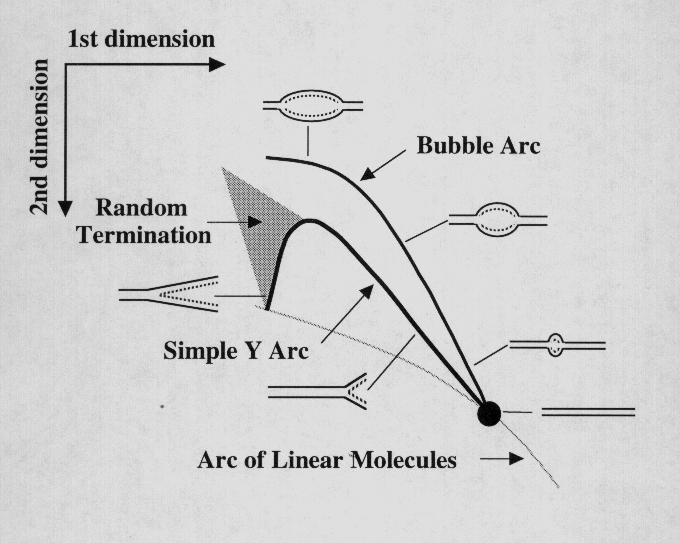

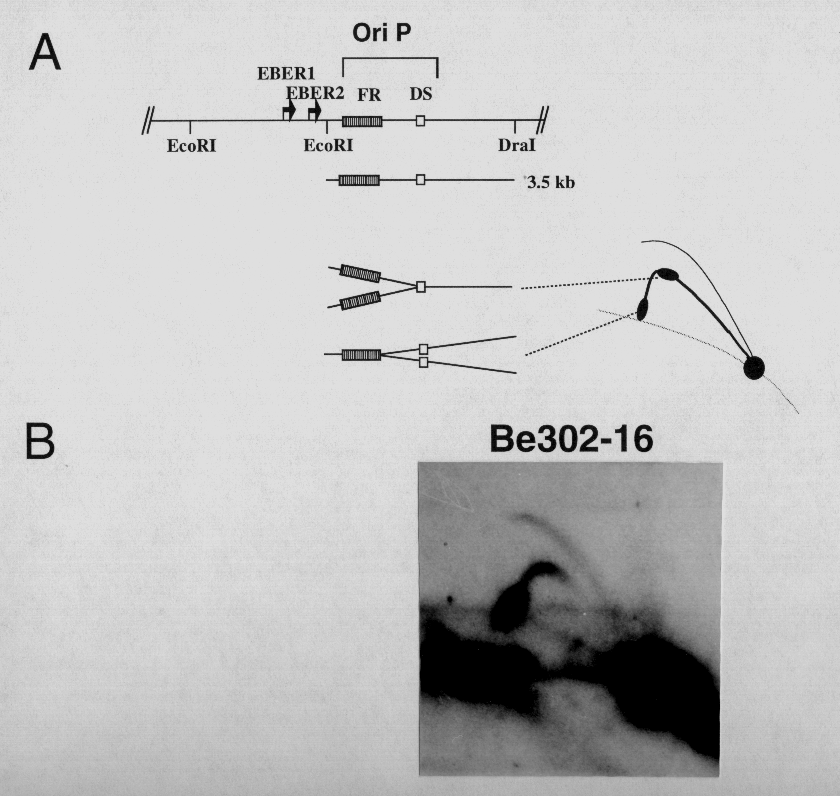

Supplement: Figure S2 — The diagram on top explains the migration pattern of different replication intermediates expected from 2-D gel analysis, including Y-arcs and bubble arcs. The dark spot in the lower right is the position where non-replicating monomer-length molecules migrate. Non-replicating linear molecules of different sizes migrate along the arc indicated. Three classes of replicating intermediates are shown in the diagram: The bubble arc showing internal initiation site, the simple Y-arc indicating that the fragment is passively replicated by single fork originated outside the fragment and random termination showing two replication forks converge at multiple sites across the region. (A) Analysis of replicative intermediates for the cell line Be302-16 latently infected with a mini EBV genome having 71% of the entire EBV sequence deleted. EcoRI and DraI digested nuclear matrix associated DNA (75 to 100 µg) were enriched for replication intermediate through BND column chromatography. After 2-D gel electrophoresis and southern transfer the membranes were hybridized to a 1 kb EcoRI-MluI fragment containing the FR region (B95-8 coordinates #7316 to #8315). On top a scheme of oriP is shown including a diagram of the 2-D gel pattern obtained with this cell line. (B) Autoradiogram of the hybridized blot. Here we did not see any random termination or accumulation of small mass close to 1× spot indicating that not many forks are coming from outside the segment analyzed, but we observed strong initiation from oriP. (DOC) [file pone.0018609.s002.doc]
