# Supplementary material for: The Dyad Symmetry Element of Epstein-Barr Virus Is a Dominant but Dispensable Replication Origin
Source: PLoS One. 2011 May 16;6(5):e18609. doi: 10.1371/journal.pone.0018609 (PMC3095595; doi:10.1371/journal.pone.0018609)
Supplement: Table S1 — Establishment efficiency of p2908 and p2910ΔDS. 5×10^5^ cells were transfected with 2 µg purified mini-EBV DNA. At 2 days posttransfection, the percentage of EGFP-positive cells was determined and duplicates of 104 and 103 cells were plated per 15-cm dish in media containing 45 µg/ml hygromycin B. After selection for 2–3 weeks, the drug-resistant colonies were counted. These data allowed the calculation of the establishment efficiency using the equation: (No. of drug-resistant colonies/with No. of EGFP-positivel transfected cells)×100%. a) Numbers reflect the average of 2 dilutions of cells plated. b)Transfection efficiency is based upon the percentage of EGFP-positive cells present at 2 days posttransfection. (DOC) [file pone.0018609.s003.doc]

**Table S1**

|  | Avg No. drug-resistant colonies^a^ | Transfection efficiency^b^  (2 experiments) | Establishment efficiency (%) |
| --- | --- | --- | --- |
| p2908 wt | 23 (10^3^); 29 (10^4^) | 37 %; 41% | 6.2; 7.1 |
|  |  |  |  |
| p2910ΔDS | 21 (10^3^); 24 (10^4^) | 43%; 39 % | 4.9; 6.2 |
